# Epicardial adipose tissue dispersion at CT and recurrent atrial fibrillation after pulmonary vein isolation

**DOI:** 10.1007/s00330-023-10498-2

**Published:** 2024-01-10

**Authors:** Adrian Thomas Huber, Severin Fankhauser, Severin Wittmer, Laureve Chollet, Anna Lam, Jens Maurhofer, Antonio Madaffari, Jens Seiler, Helge Servatius, Andreas Haeberlin, Fabian Noti, Nicolas Brugger, Hendrik von Tengg-Kobligk, Christoph Gräni, Laurent Roten, Hildegard Tanner, Tobias Reichlin

**Affiliations:** 1 Department of Diagnostic, Interventional and Pediatric Radiology, Inselspital University Hospital, University of Bern, Freiburgstrasse, 3010 Bern, Switzerland; 2 Department of Radiology and Nuclear Medicine, Lucerne Cantonal Hospital, University of Lucerne, Lucerne, Switzerland, Lucerne, Switzerland; 3 Department of Cardiology, Inselspital University Hospital, University of Bern, Bern, Switzerland

**Keywords:** Multidetector computed tomography, Adipose tissue, Atrial fibrillation, Catheter ablation, Coronary vessels

## Abstract

**Objectives:**

Epicardial adipose tissue (EAT) remodeling is associated with atrial fibrillation (AF). Left atrial (LA) EAT dispersion on cardiac CT is a non-invasive imaging biomarker reflecting EAT heterogeneity. We aimed to investigate the association of LA EAT dispersion with AF recurrence after pulmonary vein isolation (PVI).

**Methods:**

In a prospective registry of consecutive patients undergoing first PVI, mean EAT attenuation values were measured on contrast-enhanced cardiac CT scans in Hounsfield units (HU) within low (− 195 to − 45 HU) and high (− 44 to − 15 HU) threshold EAT compartments around the left atrium (LA). EAT dispersion was defined as the difference between the mean HU values within the two EAT compartments. Continuous variables were compared between groups using the Mann–Whitney *U* test and cox proportional hazard models were used to calculate hazard ratios of predictors of 1-year AF recurrence.

**Results:**

A total of 208 patients were included, 135 with paroxysmal AF and 73 with persistent AF. LA EAT dispersion was significantly larger in patients with persistent compared to paroxysmal AF (52.6 HU vs. 49.9 HU; *p* = 0.001). After 1 year of follow-up, LA EAT dispersion above the mean (> 50.8 HU) was associated with a higher risk of AF recurrence (HR 2.3, 95% CI 1.5–3.6; *p* < 0.001). It retained its predictive value when corrected for age, sex, body mass index, LA volume, and AF type (HR 2.8, 95% CI 1.6–4.6; *p* < 0.001).

**Conclusion:**

A larger LA EAT dispersion on contrast-enhanced cardiac CT scans, reflecting EAT heterogeneity, is independently associated with AF recurrence after PVI.

**Clinical relevance statement:**

Based on LA EAT dispersion assessment, a more accurate risk stratification and patient selection may be possible based on a pre-procedural cardiac CT when planning PVI.

**Key Points:**

*• Epicardial adipose tissue (EAT) remodeling is associated with atrial fibrillation (AF).*

*• A larger left atrial EAT dispersion in a pre-procedural cardiac CT was associated with a higher 1-year AF recurrence risk after pulmonary vein isolation.*

*• A pre-procedural cardiac CT with left atrial EAT dispersion assessment may provide a more accurate risk stratification and patient selection for PVI.*

**Supplementary Information:**

The online version contains supplementary material available at 10.1007/s00330-023-10498-2.

## Introduction

Epicardial adipose tissue (EAT) is metabolically active [1] and interacts with the neighboring left atrial (LA) myocardium through secretion of adipo-cytokines and reactive oxidative species [2]. LA EAT and LA myocardial remodeling are closely linked to each other and represent anatomic substrates of atrial fibrillation (AF) [3, 4].

Non-invasive imaging with cardiac CT allows EAT characterization by measurement of the mean x-ray attenuation in Hounsfield units (HU) [5]. A lower EAT attenuation has been associated with structural [6] and electrical LA remodeling [7] and AF recurrence [8]. A lower EAT attenuation in CT may be explained by EAT adipocyte hypertrophy with larger lipid droplets, reflecting an unfavorable metabolic EAT activity [5]. This is fostered by ex vivo histology analysis showing a positive association of EAT adipocyte diameter with the degree of EAT fibrotic remodeling and body mass index (BMI) in AF patients [9]. Conversely, some other studies found a higher EAT attenuation in patients with AF recurrence when measured on contrast-enhanced cardiac CT scans, as an indicator of EAT inflammation [10]. All those observations may be explained by an increasing LA EAT heterogeneity with co-existence of EAT adipocyte hypertrophy, inflammation, and fibrosis. In addition, the measured CT EAT attenuation values are dependent on patient ethnicity, sex, and BMI [11], as well as different threshold values used for EAT segmentation, with lower threshold values ranging between − 195 and − 190 HU and upper threshold values ranging between − 45 and − 15 HU [10, 12, 13].

Based on previously published threshold values, a low threshold EAT compartment may be segmented between − 45 and − 195 HU [10, 12]. However, based on other investigations, a higher threshold of − 15 HU may be appropriate, especially when investigating contrast-enhanced CT scans [13, 14]. Instead of calculating mean EAT attenuation in the total EAT compartment, EAT attenuation may be calculated separately in two adjacent high (− 15 to − 45 HU) and low (− 45 to − 195 HU) threshold EAT compartments. EAT dispersion may then be calculated as the difference of attenuation between the high and low threshold EAT and represents a potential non-invasive imaging surrogate of EAT heterogeneity. We hypothesized that a larger EAT dispersion reflects co-existing adipocyte hypertrophy, inflammation, and fibrosis during EAT remodeling and is associated with an adverse outcome in AF patients. The aim of this study was to investigate the association of LA EAT dispersion with AF phenotype and with AF recurrence after pulmonary vein isolation (PVI).

## Methods

### Study population

This was a secondary analysis of prospectively collected data [14]. Consecutive patients undergoing a first AF ablation by means of pulmonary vein isolation (PVI) with a pre-procedural CT scan at the Inselspital, Bern University Hospital, Bern, Switzerland, were prospectively enrolled into an institutional registry. The registry was approved by the Bern cantonal ethics committee and the study was carried out in accordance with the principles of the Declaration of Helsinki. The authors had full access to and take full responsibility for the integrity of the data.

For the purpose of the present analysis, the following patients were excluded: those declining consent, patients without pre-procedural CT scan, those with supplemental ablations in the left atrium in addition to pulmonary vein isolation (PVI), and patients with a history of any previous LA procedure.

### Baseline evaluation

All patients underwent pre-procedural clinical evaluation including detailed medical history and standard blood tests. Paroxysmal AF was defined as AF that terminates spontaneously or with intervention within 7 days of onset. Persistent AF was defined as AF that continuously sustained beyond 7 days [15]. Transthoracic echocardiography was performed at baseline prior to the procedure according to the guidelines from the American Society of Echocardiography [16] to assess left ventricular ejection fraction (LVEF) and LA diameter in the parasternal long axis.

### Computed tomography protocol

Pre-procedural cardiac CT scans were performed on a Siemens Somatom Definition Flash CT (Siemens Healthineers). Both a noncontrast and a contrast-enhanced scan were acquired in inspiratory breath-hold. All acquisitions were electrocardiogram-triggered using either a prospectively triggered acquisition with a high pitch of 3.2 in patients with irregular heart rhythm or a retrospectively triggered acquisition in patients with regular heartbeats, both triggered on 70% of the RR interval. All scans were acquired with a collimation of 128 × 0.6 mm and a gantry rotation time of 0.28 s. Noncontrast CT was acquired for calcium scoring as a part of the patient’s standard of care imaging with 120 kVp and 80 mAs, while contrast-enhanced scans were acquired with 120 reference kV and 250 reference mAs, using the CARE dose mode. Intravenous contrast medium was injected with a CT Exprès contrast media delivery system (Bracco Diagnostics Inc.). Contrast-enhanced scans were acquired after injection of 90-mL Ultravist 370 (Bayer Healthcare) into the left brachial vein with a flow rate of 4.5 mL/s, followed by a 20-mL saline chaser with a flow rate of 4.5 mL/s. For the contrast-enhanced scans, bolus-tracking was used, triggered on the ascending aorta. All noncontrast CT images were reconstructed in 3-mm axial sections with a reconstruction kernel B35f and contrast-enhanced images were reconstructed in 0.75-mm axial sections with a reconstruction kernel I30f using a sinogram affirmed iterative reconstruction (SAFIRE) algorithm.

### Image analysis

**Fig. 1:**
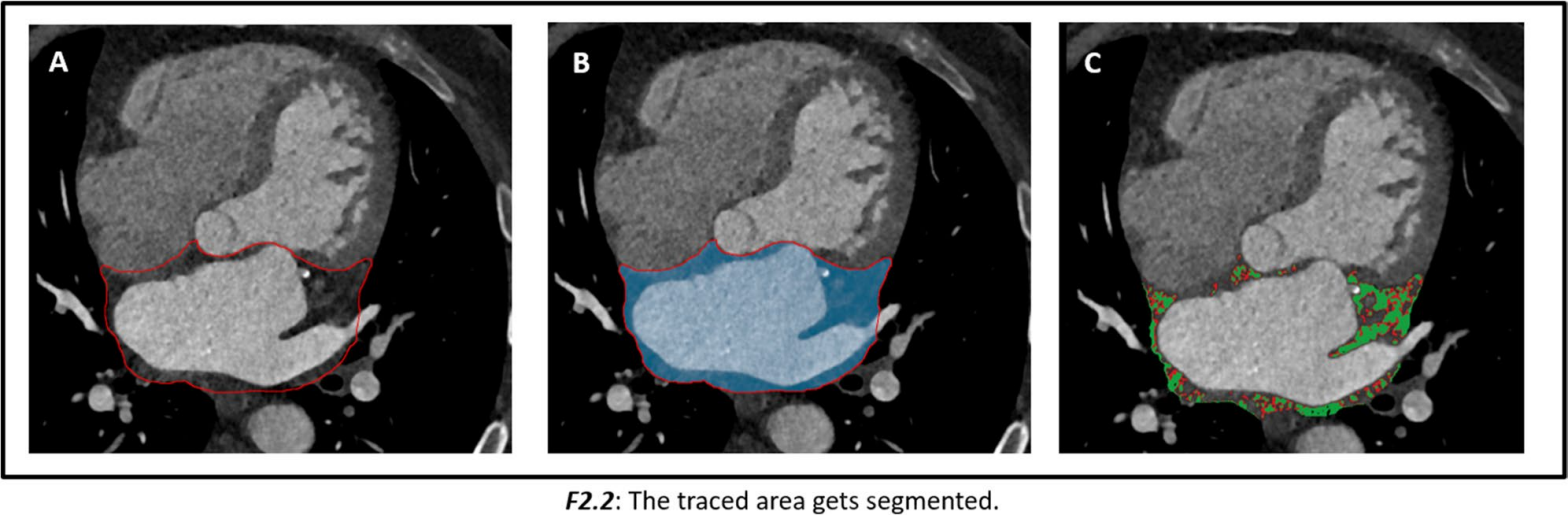
Epicardial adipose tissue (EAT) dispersion calculation on a contrast-enhanced CT scan around the left atrium (LA). **A** The LA EAT within the pericardium was segmented between the coronary sinus, the superior margin of the left atrial appendage, the mitral valve, and the interatrial septum (red line). **B** Voxels within the included volume (blue color) were analyzed and separated in a low (voxels in green between − 195 and − 45 HU) and high (voxels in red between − 45 and − 15 HU) threshold LA EAT compartment (**C**)

**Fig. 2:**
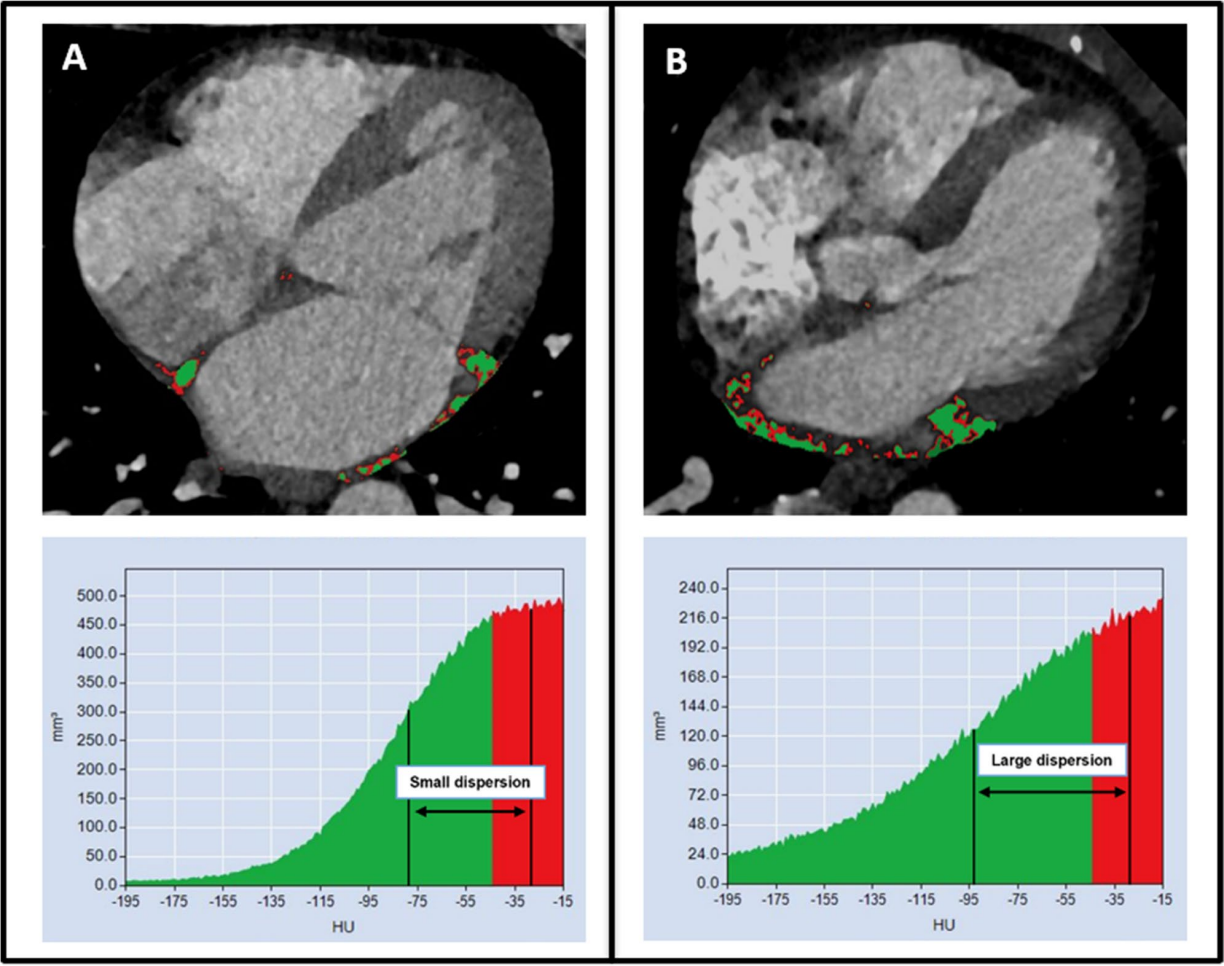
Examples of two patients with left atrial (LA) epicardial adipose tissue (EAT) dispersion calculation. Patient **A** with no AF recurrence had a small LA EAT dispersion, defined as the difference between the mean attenuation of the low (green) and high (red) threshold EAT compartment. Patient **B** with AF recurrence had a high LA EAT dispersion

Image analysis was performed on the contrast-enhanced cardiac CT scans on a dedicated workstation (Aquarius Workstation version 4.4.13.P6, TeraRecon). Contours were drawn manually around the LA EAT on 3–5 slices and then interpolated automatically between those slices to assess the whole left atrial EAT. The automatically interpolated layers were reconsidered and manually adjusted to the defined anatomical boarders. Interpolation errors were thereby minimized. The left atrial EAT was defined as the EAT within the pericardium between the coronary sinus and the superior margin of the left atrial appendage on the left, and the pulmonary artery bifurcation on the right. The interatrial septum was included, while the mitral valve annulus represented the anterior margin of the segmented LA EAT volume (Fig. 1). In addition, EAT was automatically segmented in a cylindric volume 4 mm around the right coronary artery (RCA), the left anterior descending artery (LAD), and the left circumflex artery (LCX), starting 1 cm from the origin of the RCA and directly from the origin of the LAD and LCX over a distance of 4 cm (Supplemental Fig. 1). All EAT densities were adjusted for a 120 kVp tube voltage as published before [17, 18]. Mean EAT attenuation was measured in the low threshold EAT compartment (threshold − 195 and − 45 HU), and in the high threshold EAT compartment (threshold between − 44 and − 15 HU), as well as in the complete EAT compartment (threshold between − 195 and − 15 HU). EAT dispersion was defined as the difference between the mean attenuation of the low and high threshold EAT compartment. Examples of two patients with LA EAT dispersion calculation are shown in Fig. 2. LA enhancing EAT (e-EAT) was calculated as the LA EAT volume difference between the noncontrast and contrast-enhanced scan divided by the total LA EAT volume on the noncontrast-enhanced scan, as previously reported [14]. The coronary artery calcium (CAC) score was measured on the noncontrast scans, using a threshold of 130 HU to calculate the Agatston score. LA EAT was measured on both the noncontrast and contrast-enhanced scans for LA e-EAT calculation. LA EAT dispersion and all other CT segmentations and measurements were performed in contrast-enhanced scans only.

### Reproducibility, post-processing time, and dose estimate

A total of 20 cases were randomly selected and reassessed by the same reader with a washout period of 3 months between the assessments (S.F.; 1 year of experience in cardiac imaging) for intra-rater reproducibility measurements, as well as by a second reader (A.T.H.; 11 years of experience in cardiac imaging) for inter-rater reproducibility measurements. Intraclass correlation coefficients (ICC) were calculated for LA EAT dispersion, LAD EAT dispersion, RCA EAT dispersion, and LCX EAT dispersion, using a two-way mixed model. An ICC of below 0.5 was defined as poor agreement, 0.5–0.75 as moderate agreement, 0.75–0.90 as good agreement, and above 0.90 as excellent agreement [19]. The post-processing time used for LA EAT segmentation and radiation dose were recorded.

### Ablation procedures

Procedures were performed as per the standard of care protocol in our institution and in accordance to current guidelines. For a minority of high-risk patients, general anesthesia with endotracheal intubation was used.

### Follow-up

Follow-up included 7-day Holter electrocardiograms at 3, 6, and 12 months and in case of symptoms. The primary endpoint was recurrence of an atrial arrhythmia between days 90 and 365 after ablation. Atrial arrhythmia was defined as AF, atrial flutter, or atrial tachycardia lasting > 30 s.

### Statistical analysis

Categorical variables are reported as numbers and percentages, and comparisons between groups were performed using Fisher’s exact tests. Continuous variables are reported as medians and interquartile ranges (IQR), and comparisons between groups were performed using the Mann–Whitney *U* test. Recurrence-free survival curves were constructed using the Kaplan–Meier method. Cox proportional hazard models were used to calculate hazard ratios and 95% CIs of predictors of 1-year recurrence. Continuous variables were dichotomized at mean values. Multivariable models were adjusted for sex, age, BMI, AF type, and CT LA volume index. A *p*-value of < 0.05 was considered the threshold for statistical significance. Statistical analyses were performed using the SPSS Statistics, Version 25 (IBM Corp. 2017) software.

## Results

### Study population

Among 271 consecutive patients that underwent AF ablation with pre-procedural CT, 208 individuals met the inclusion criteria (median age, 64 [IQR, 57–71]; 156 (75%) men; Table 1, Supplemental Fig. 2). Paroxysmal AF was present in 135/208 (65%) and persistent AF in 73/208 (35%) of the patients. Age and sex did not differ significantly between patients with paroxysmal AF and patients with persistent AF. Patients with persistent AF had a higher body mass index, prevalence of hypertension, heart failure, and NT-pro-BNP values.

**Table 1:** Baseline characteristics of the study population

	All patients (*n* = 208)	Paroxysmal AF (*n* = 135)	Persistent AF (*n* = 73)	*p*-value
Age, years	64 (57–71)	63 (56–71)	64 (58–71)	.49
No men (%)	156 (75%)	97 (72%)	59 (81%)	.25
BMI, kg/m^2^	27 (25–31)	26 (24–30)	29 (25–35)	< .001
AF duration, months	14 (4–48)	10 (3–48)	17 (5–44)	.47
AF duration, if persistent, months	N/A	N/A	6 (3–12)	N/A
History				
Arterial hypertension	127 (61%)	74 (55%)	53 (73%)	.01
Coronary artery disease	33 (16%)	19 (14%)	14 (19%)	.33
Heart failure	48 (23%)	13 (10%)	35 (48%)	< .001
Diabetes	15 (7%)	9 (7%)	6 (8%)	.59
Prior TIA or stroke	16 (8%)	13 (10%)	3 (4%)	.18
Prior hospitalization for AF	98 (47%)	62 (46%)	36 (49%)	.67
CHA_2_DS_2_-VASC score	2 (1–3)	2 (1–3)	2 (1–3)	.03
Oral anticoagulation	194 (93%)	123 (90%)	72 (99%)	.02
Amiodarone	74 (36%)	40 (30%)	34 (47%)	< .01
NT-proBNP, ng/L	445 (179–1014)	274 (101–568)	897 (535–1855)	< .001
eGFR	78 (64–91)	79 (68–93)	75 (61–89)	.06
Echocardiography				
LVEF (%)	57 (50–60)	60 (55–60)	52 (40–55)	< .001
LVEDD, mm	49 (44–54)	48 (42–53)	50 (45–56)	.03
LA diameter (mm)	44 (38–49)	43 (37–47)	46 (41–53)	< .01
CT				
LA volume, index (mL/m^2^)	66 (53–84)	59 (51–73)	84 (70–98)	< .001
LA EAT volume, index (mL/m^2^)	13 (10, 18)	12 (9, 16)	16 (11, 23)	< .01
LA e-EAT (%)	34 (24, 43)	32 (22, 42)	37 (26, 46)	.09
Calcium score total	199 (63–527)	199 (66–487)	206 (61–781)	.54

Median values are shown with lower and upper interquartile range for continuous variables and frequencies with percentages for categorical variables. *p*-values were calculated using Mann–Whitney *U* test or Fishers exact test, as appropriate, to evaluate differences between paroxysmal and persistent AF. Total EAT was segmented between − 195 and − 15 HU. Low attenuation EAT was segmented between − 195 and − 45 HU. High attenuation EAT was segmented between − 44 and − 15 HU. EAT dispersion was defined as the difference between the low attenuation and high attenuation mean value. LA enhancing EAT (e-EAT) was calculated as the LA EAT volume difference between the noncontrast and contrast-enhanced scan divided by the total LA EAT volume on the noncontrast-enhanced scan, as previously reported (14)

*BMI*, body mass index; *AF*, atrial fibrillation; *TIA*, transitory ischemic attack; *INR*, international normalized radio; *eGFR*, estimated glomerular filtration rate; *NT-proBNP*, N-terminal pro b-type natriuretic peptide; *LVEF*, left ventricular ejection fraction; *LA*, left atrium

### Association of cardiac dimensions, EAT attenuation, enhancement dispersion, and AF type

In patients with persistent AF, the left ventricle (LV) was enlarged and left ventricular ejection fraction (LVEF) was slightly reduced. The left atrium (LA) was enlarged both in echocardiography and as measured by CT LA volumetry (Table 1). CAC score was not significantly different between patients with paroxysmal and persistent AF. The association of CT EAT attenuation and dispersion with AF type is shown in Table 2. LA EAT attenuation was significantly lower in patients with persistent than in patients with paroxysmal AF in the total EAT, as well as in the low and high threshold EAT. LA e-EAT was not significantly higher in patients with persistent AF. When measured around the coronary arteries, EAT was significantly lower in the low threshold EAT in patients with persistent AF, but not in the high threshold and total EAT. EAT dispersion was significantly larger in patients with persistent AF around the LA, the RCA, and the LCX, but not the LAD.

**Table 2:** Association of CT EAT attenuation, dispersion, and AF phenotype

	Paroxysmal AF (*n* = 135)	Persistent AF (*n* = 73)	*p*-value
EAT attenuation − 15 to − 195 HU			
LA, HU	− 59.1 (− 63.3, − 55.3)	− 62.0 (− 66.0, − 57.5)	.01
RCA, HU	− 71.5 (− 77.1, − 62.7)	− 71.5 (− 80.1, − 64.5)	.39
LCX, HU	− 56.5 (− 62.0, − 51.4)	− 58.0 (− 67.4, − 52.9)	.13
LAD, HU	− 62.3 (− 68.2, − 57.4)	− 63.7 (− 67.4, − 56.4)	.92
EAT attenuation − 15 to − 45 HU			
LA, HU	− 26.3 (− 26.7, − 26.0)	− 26.6 (− 29.2, − 26.2)	< .01
RCA, HU	− 26.9 (− 27.5, − 26.4)	− 27.1 (− 29.7, − 26.5)	.06
LCX, HU	− 26.7 (− 27.2, − 26.2)	− 26.8 (− 29.2, − 26.3)	0.20
LAD, HU	− 27.0 (− 27.6, − 26.7)	− 27.0 (− 29.6, − 26.6)	0.37
EAT attenuation − 45 to − 195 HU			
LA, HU	− 76.6 (− 79.8, − 73.9)	− 79.2 (− 83.1, − 75.4)	< .001
RCA, HU	− 74.4 (− 81.5, − 69.3)	− 78.6 (− 84.5, − 72.4)	< .01
LCX, HU	− 72.2 (− 76.7, − 67.9)	− 75.3 (− 82.6, − 69.8)	.02
LAD, HU	− 75.5 (− 80.8, − 71.6)	− 78.7 (− 82.9, − 74.3)	.04
EAT dispersion			
LA, HU difference	49.9 (52.7, 47.0)	52.6 (55.5, 48.4)	< 0.001
RCA, HU difference	46.9 (54.0, 41.8)	51.2 (57.7, 43.9)	0.02
LCX, HU difference	45.4 (49.6, 41.2)	47.0 (54.3, 42.4)	0.05
LAD, HU difference	48.2 (52.7, 43.9)	51.1 (54.0, 46.0)	0.08

Median values are shown with lower and upper interquartile range in parenthesis. The *p*-values were calculated using the Mann–Whitney *U* test. Total EAT was segmented between − 195 and − 15 HU. Low attenuation EAT was segmented between − 195 and − 45 HU. High attenuation EAT was segmented between − 45 and − 15 HU. EAT dispersion was defined as the difference between the low attenuation and high attenuation mean value

*AF*, atrial fibrillation; *HU*, Hounsfield units; *LA*, left atrium; *RCA*, right coronary artery; *LCX*, left circumflex artery; *LAD*, left anterior descending artery

### Impact of EAT dispersion on outcome after PVI

After 1 year of follow-up, the recurrence rate of AF was 76/208 (37%). There was no significant difference of AF recurrence in patients undergoing radiofrequency ablation (32/72, 44%) and patients undergoing cryoablation (44/136, 32%; *p* = 0.10).

**Fig. 3:**
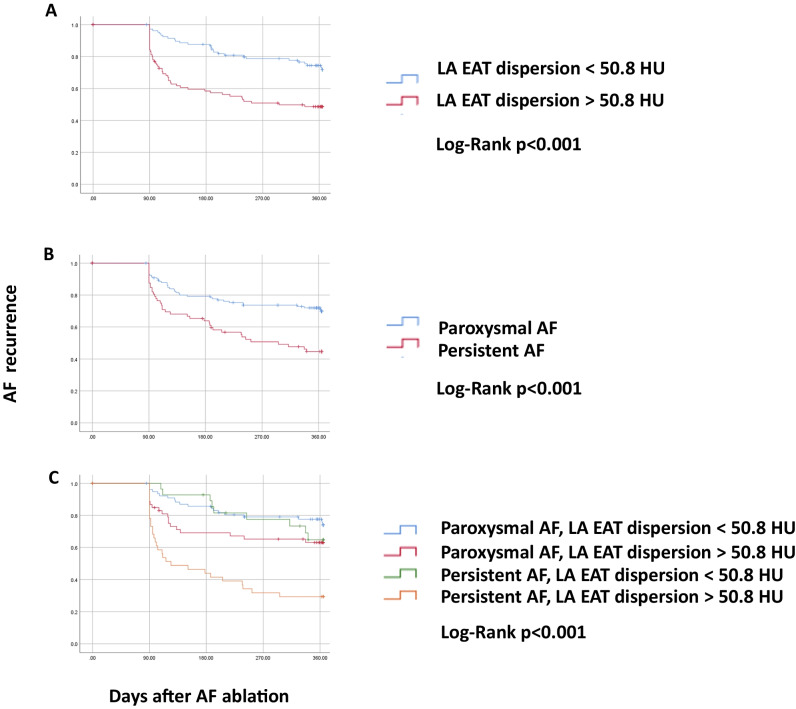
Kaplan–Meier curves showing AF recurrence in patients with (**A**) LA EAT dispersion, (**B**) AF phenotype, and (**C**) a combination of AF phenotype with LA EAT dispersion. AF recurrence was significantly more frequent in patients with LA EAT dispersion > 50.8 HU (red curve in **A**) and in patients with persistent AF (red curve in **B**). Patients with LA EAT dispersion < 50.8 and paroxysmal AF had the lowest AF recurrence rate of 22% (blue curve in **C**), while patients with LA EAT dispersion > 50.8 and persistent AF had the highest AF recurrence rate of 71% (*p* < 0.001, orange curve in **C**). AF, atrial fibrillation; LA, left atrial; EAT, epicardial adipose tissue

Kaplan–Meier curve analysis showed that patients with LA EAT dispersion above the mean had a higher AF recurrence rate (51%) than patients with LA EAT dispersion below the mean (28%, *p* < 0.001; Fig. 3(A)). Similarly, patients with persistent AF had a higher AF recurrence rate (55%) than patients with paroxysmal AF (28%, *p* < 0.001; Fig. 3(B)). The highest AF recurrence rate (71%) was observed in patients with both persistent AF and LA EAT dispersion above the mean. Patients with persistent AF/LA EAT dispersion below the mean and paroxysmal AF/LA EAT dispersion above the mean had similar AF recurrence rates (35% and 37%, respectively), while the lowest AF recurrence rate was seen in patients with paroxysmal AF and LA EAT dispersion below the mean (22%, *p* < 0.001; Fig. 3(C)).

In univariate analysis, persistent AF was a strong predictor of AF recurrence after 1 year, as was LA e-EAT, previous stroke or TIA, a reduced LVEF, increased LA diameter, and enlarged CT LA volume index. All other clinical parameters were not predictive of AF recurrence (Table 3). However, LA EAT dispersion was the strongest predictor with a 2.3 times higher risk for AF recurrence (HR 2.3, 95% CI 1.5–3.6; *p* < 0.001). In multivariate analysis, LA EAT dispersion retained its predictive value of AF recurrence when corrected for sex, age, BMI, LA e-EAT, LA EAT volume, LA volume, and AF type (HR 2.7, 95% CI 1.6–4.5; *p* < 0.001), as shown in Table 4.

**Table 3:** Univariate Cox regression analysis for AF recurrence after 1 year

	Hazard ratio	95% confidence interval	*p*-value
Male sex	1.1	0.6–1.7	.84
Age, ≥ 70 years old	0.9	0.6–1.5	.79
BMI, ≥ 30 kg/m^2^	1.2	0.7–1.9	.55
Arterial hypertension	1.2	0.7–1.8	.54
Coronary heart disease	1.0	0.6–1.8	.98
Heart failure	1.3	0.8–2.2	.24
Diabetes	0.8	0.3–2.0	.62
Previous stroke or TIA	2.2	1.1–4.5	.02
Persistent AF	2.2	1.4–3.5	< .001
LVEF, < 55%	1.7	1.1–2.7	.02
LA diameter, mm *	1.6	1.0–2.6	.08
CT LA volume index (mL/m^2^)*	2.2	1.4–3.5	< .01
CT LA EAT volume, index (mL/m^2^)	1.0	1.0–1.0	.10
LA e-EAT (%)	2.4	1.5–3.9	< .001
A. EAT attenuation (− 195 to − 15 HU) **			
LA, HU	1.6	1.0–2.5	.06
RCA, HU	1.5	0.9–2.4	.09
LCX, HU	1.8	1.1–3.0	.02
LAD, HU	1.2	0.8–2.0	.35
B. EAT dispersion *			
LA, HU	2.3	1.5–3.6	< .001
RCA, HU	2.0	1.2–3.2	< .01
LCX, HU	2.1	1.2–3.4	< .01
LAD, HU	1.5	0.9–2.4	.09

Univariate Cox regression analysis to predict AF recurrence 1 year after pulmonary vein isolation (PVI). Total EAT was segmented between − 195 and − 15 HU. Low attenuation EAT was segmented between − 195 and − 45 HU. High attenuation EAT was segmented between − 44 and − 15 HU. EAT dispersion was defined as the difference between the low attenuation and high attenuation mean value. LA enhancing EAT (e-EAT) was calculated as the LA EAT volume difference between the noncontrast and contrast-enhanced scan divided by the total LA EAT volume on the noncontrast-enhanced scan, as previously reported (14)

*AF*, atrial fibrillation; *BMI*, body mass index; *TIA*, transitory ischemic attack; *LFEF*, left ventricular ejection fraction; *LA*, left atrium; *HU*, Hounsfield units; *RCA*, right coronary artery; *LCX*, left circumflex artery; *LAD*, left anterior descending artery. *ref = below the mean, **ref = above the mean

**Table 4:** Multivariate cox regression analysis to predict AF recurrence after 1 year

	Hazard ratio	95% confidence interval	*p*-value
LA EAT dispersion, HU	2.6	1.5–4.4	< 0.001
LA e-EAT, %	2.2	1.2–4.0	< .01
LA volume index mL/m^2^	1.3	0.8–2.4	.30
CT LA EAT volume, index (mL/m^2^)	1.0	1.0–1.1	.24
Persistent AF	1.8	1.1–2.9	.02
Age, ≥ 70 years old	0.8	0.4–1.3	.32
Male sex	0.9	0.6–1.7	.95
BMI, ≥ 30 kg/m^2^	0.6	0.3–1.0	.05

Multivariate Cox regression analysis to predict atrial fibrillation AF recurrence 1 year after pulmonary vein isolation (PVI)

Epicardial adipose tissue (EAT) dispersion was defined as the difference between the low attenuation and high attenuation mean EAT value. LA volume index mL/m^2^ was calculated on the contrast-enhanced scan. LA enhancing EAT (e-EAT) was calculated as the LA EAT volume difference between the noncontrast and contrast-enhanced scan divided by the total LA EAT volume on the noncontrast-enhanced scan, as previously reported (14)

*AF*, atrial fibrillation; *BMI*, body mass index; *LA*, left atrium; *LAA*, left atrial appendage; *EAT*, epicardial adipose tissue; *NE-EAT*, non-enhancing epicardial adipose tissue

RCA EAT dispersion (HR 2.0, 95% CI 1.2–3.2; *p* < 0.001) and LCX EAT dispersion (HR 2.1, 95% CI 1.2–3.4; *p* < 0.01) were predictive of AF recurrence as well, but with a lower performance than LA EAT dispersion. LAD EAT dispersion did not reach statistical significance (HR 1.5, 95% CI 0.9–2.4; *p* = 0.09; Table 3).

### Reproducibility, post-processing time, and dose estimate

Intra-rater reproducibility was excellent for LA EAT dispersion (0.96; *p* < 0.001), LAD EAT dispersion (0.98; *p* < 0.001), and RCA EAT dispersion (0.96; *p* < 0.001) and good for LCX EAT dispersion (0.86; *p* < 0.001). The median radiation dose for the whole exam was 1.4 mSv (interquartile range 1.2–2.8 mSv). Median post-processing time for LA EAT segmentation was 5 min (interquartile range 4–6 min).

## Discussion

This study underlines the association between EAT remodeling and the risk of AF recurrence after PVI. Patients with AF recurrence after PVI have a higher EAT metabolic activity [14, 20] and a larger EAT volume [21–23], which in turn has been associated with lower EAT attenuation values [24]. Our findings of lower CT EAT attenuation values in patients with AF recurrence after PVI and in patients with persistent AF are therefore in line with the findings of other research groups [8]. CT EAT dispersion is a new imaging biomarker based on the concept that metabolically active EAT enlarges with ongoing heterogeneous processes of adipocyte hypertrophy [25], EAT inflammation, and fibrosis [26]. CT EAT dispersion represents a non-invasive imaging biomarker of EAT heterogeneity and is larger in patients with structural and electrical EAT remodeling including those with persistent AF compared to paroxysmal AF.

Peri-coronary EAT dispersion around the LCX and the RCA was found as well, even though less performant than LA EAT dispersion. One possible explanation for this observation is that more EAT changes may be observed around the LAD in patients with coronary artery disease, while in atrial fibrillation, EAT changes may be more localized to the peri-atrial EAT, due to local electrophysiological and biochemical interactions between the LA myocardium and the surrounding EAT in patients with atrial fibrillation, as shown in studies investigating coronary [27–29], valvular [30], and cardiometabolic disease [31, 32]. Beyond its value in AF patients undergoing PVI, EAT dispersion might be an interesting non-invasive imaging biomarker also in other cardiovascular patients, as lower EAT attenuation values have been associated with unfavorable cardiometabolic risk profiles [33], obstructive coronary artery disease [34], and myocardial infarction [12].

An important concept corroborated by this study is that the obtained EAT attenuation values are highly dependent on the chosen threshold values, possibly explained by heterogeneous processes of adipocyte hypertrophy, inflammation, and fibrosis, allocating an EAT voxel to a higher or lower position in the EAT attenuation spectrum. EAT voxels with many adipocytes may be allocated to the lower EAT attenuation compartment and therefore more prone to a decrease of attenuation in case of adipocyte hypertrophy. Vice versa, EAT voxels with less adipocytes, but inflammation and fibrosis, may be allocated to the higher EAT attenuation compartment and prone to an increase of attenuation in case of EAT inflammation and fibrosis. EAT dispersion measures the difference between the high- and low-density EAT compartment und therefore represents an attractive non-invasive surrogate for the increasing EAT heterogeneity. However, such an association between EAT dispersion and possible co-occurring processes during structural and electrical EAT remodeling was not analyzed in the present study and warrants further investigations.

The type of AF is the single best clinical predictor of AF recurrence after PVI [35]. Interestingly, LA EAT dispersion showed a similar performance to predict AF recurrence than the type of AF. However, combination of LA EAT dispersion with the type of AF allowed to refine prediction of AF recurrence in both patients with paroxysmal and persistent AF.

This study has several limitations. First, this was a single center study with a retrospective analysis of a prospective registry. The results should therefore be externally validated in an independent patient population, using the same methods. Second, EAT attenuation and dispersion may vary between different CT vendors, and differences in the used tube current and voltage, as well as the used CT contrast medium and injection rates—although tube voltages were normalized to mitigate those confounding factors [18].

This is the first study to show that LA EAT dispersion is a predictor of AF recurrence after PVI, as a non-invasive imaging biomarker for LA EAT heterogeneity, independent from AF phenotype and other CT parameters. In combination with the AF phenotype, it allowed prognostication of 1-year AF recurrence in patients undergoing PVI, ranging from a low AF recurrence rate of 22% in patients with paroxysmal AF and LA EAT dispersion below the mean to 71% in patients with persistent AF and LA EAT dispersion above the mean. Based on those findings, a more accurate risk stratification and patient selection may be possible based on a pre-procedural cardiac CT when planning PVI.

In conclusion, a larger LA EAT dispersion on contrast-enhanced cardiac CT scans as a surrogate for EAT metabolic activity is an independent predictor of AF recurrence after PVI.

### Supplementary Information

Below is the link to the electronic supplementary material.

Supplementary file1 (PDF 155 KB)

## Abbreviations

AF: Atrial fibrillation
BMI: Body mass index
CAC: Coronary artery calcium score
EAT: Epicardial adipose tissue
HFpEF: Heart failure with preserved ejection fraction
HU: Hounsfield units
ICC: Intraclass correlation coefficients
IQR: Interquartile ranges
LA: Left atrial
LAD: Left anterior descending artery
LCX: Left circumflex artery
LVEF: Left ventricular ejection fraction
PVI: Pulmonary vein isolation
RCA: Right coronary artery

## Declarations

### Guarantor

The scientific guarantor of this publication is Prof Dr. Dr. Adrian Huber.

### Statistics and biometry

One of the authors (A.T.H.) has significant statistical expertise.

### Study subjects or cohorts overlap

Another study was published based on the same patient population, as we made transparent and cited appropriately (Huber et al, 2022, Radiology, https://doi.org/10.1148/radiol.212644). This other study analyzed the enhancement of the LA EAT between noncontrast and contrast-enhanced scans, while the present study analyzes the LA EAT dispersion solely on contrast-enhanced scans. Therefore, the two studies were different.

### Methodology

• prospective

• diagnostic or prognostic study

• performed at one institution
