# Supplementary material for: Epicardial adipose tissue dispersion at CT and recurrent atrial fibrillation after pulmonary vein isolation
Source: Eur Radiol. 2024 Jan 10;34(8):4928–38. doi: 10.1007/s00330-023-10498-2 (PMC11255050; doi:10.1007/s00330-023-10498-2)

Supplemental Figure 1. Epicardial adipose tissue (EAT) dispersion calculation on a contrast-enhanced CT scan around the right coronary artery (RCA). The EAT was measured 4 mm around the coronary arteries, starting 1 cm from the origin of the RCA (A) and directly from the origin of the LAD (B) and LCX (C) over a distance of 4 cm. Voxels within the included volume were segmented in a low (voxels in green between -195 and -45 HU) and high (voxels in red between -45 and -15 HU) threshold LA EAT compartment.

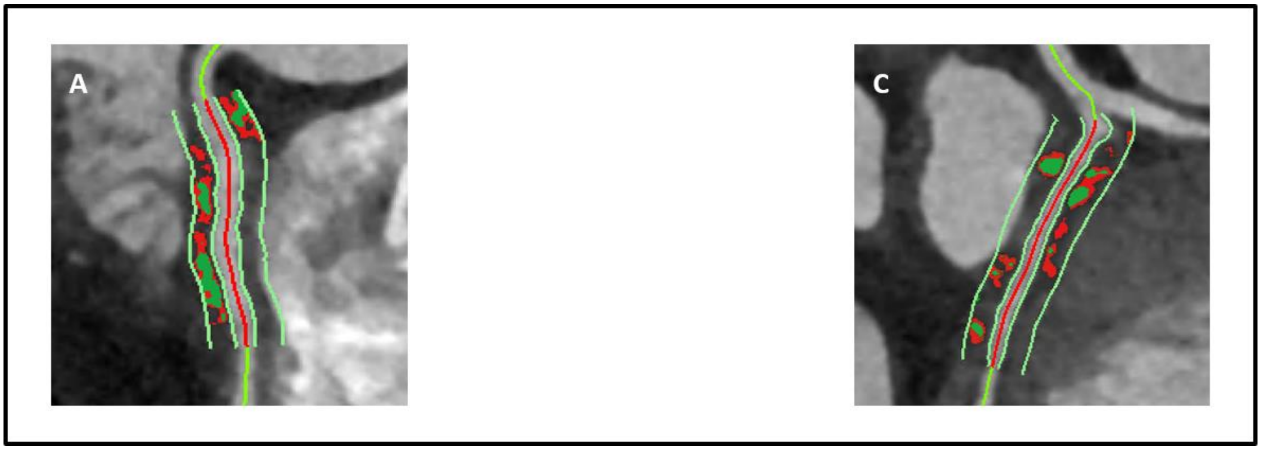

Supplemental Figure 2. Study flow diagram. A total of 271 patients undergoing a first pulmonary vein isolation (PVI) were consecutively included. 35 patients with missing informed consent, 14 patients with previous PVI and 10 patients with LA ablation in addition to PVI or a combined intervention other than PVI were excluded. After one year follow-up, 136 patients had no AF recurrence, while 76 patients had AF recurrence.

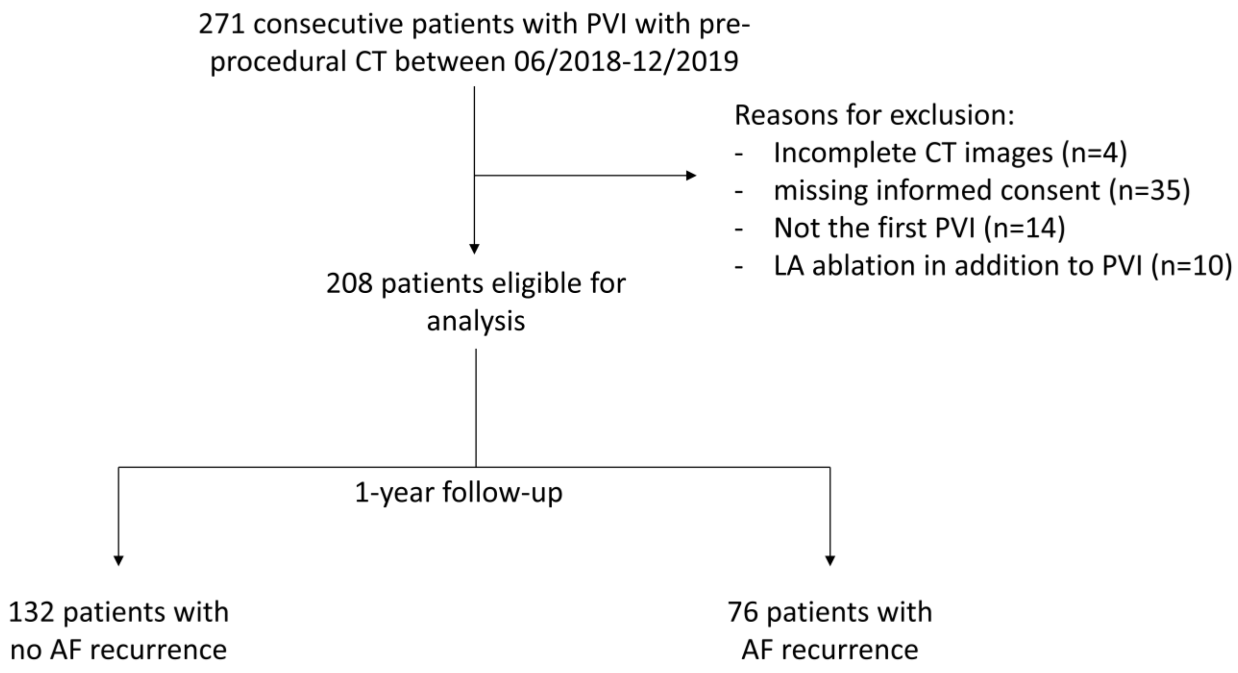

Supplement: Supplementary file 1 — Supplementary file1 (PDF 155 KB) [file 330_2023_10498_MOESM1_ESM.pdf]
